# Anxiety, Sleep Problems, and Vigorous Physical Activity: Bidirectional Associations from Early Adolescence to Early Adulthood in Swedish Adolescents

**DOI:** 10.1007/s10964-024-01980-1

**Published:** 2024-04-05

**Authors:** F. Giannotta, K. W. Nilsson, C. Åslund, S. Olofdotter, S. Vadlin, P. Larm

**Affiliations:** 1 Division of Public Health, School of Health, Care and Social Welfare, Malardalen University, Box 883, Västerås, Sweden; 2 Department of Public Health, Stockholm University, SE-106 91 Stockholm, Sweden; 3 Department of Psychology, University of Turin, via Verdi 10, 10124 Turin, Italy; 4 Centre for Clinical Research, Västmanland County Hospital Västerås, Uppsala University, S-72189 Västerås, Sweden; 5 Department of Neuroscience, Uppsala University, Uppsala, Sweden; 6 Department of Public Health and Caring Sciences, Uppsala University, Uppsala, Sweden

**Keywords:** Anxiety, Sleep problems, RI-CPLM, Physical activity, Reciprocal associations

## Abstract

Anxiety symptoms and sleep problems typically emerge during adolescence and are frequently intertwined. However, there is a dearth of knowledge concerning their reciprocal influence and whether physical activity might play a protective role in this relationship. The present study aims at filling this gap exploring also the moderating role of sex. 915 13-year-old Swedish adolescents (56% girls) answered a survey conducted four times: at ages 13 (T1), 16 (T2), 19 (T3), and 22 (T4). A random intercept cross-lagged panel model was used. At within-levels, sleep problems and anxiety symptoms had a bidirectional positive association in middle adolescence. Vigorous physical activity and anxiety symptoms showed a reciprocal negative association from middle adolescence. Vigorous physical activity and sleep problems were reciprocally associated only in late adolescence. Associations were the same for girls and boys. This study demonstrated that the relations between anxiety symptoms, sleep problems, and vigorous physical activity cannot be understood without adopting a developmental perspective and that middle adolescence is a crucial period to plan interventions to reduce anxiety symptoms and sleep problems.

## Introduction

The period spanning from early adolescence to early adulthood is crucial for the development of anxiety and sleep disorders. Anxiety issues often surface during childhood and adolescence and may progress into disorders during this period (Beesdo-Baum & Knappe, 2012). Additionally, adolescence represents a sensitive phase for sleep, characterized by changes in the circadian cycle, resulting in a delayed onset of sleep by 2–3 h compared to adults, which can conflict with societal demands such as early school start times (Gariepy et al., 2020). Conversely, a protective factor such as physical activity tends to decline from adolescence through early adulthood (Dumith et al., 2011). Despite evidence suggesting interactions in the development of anxiety symptoms and sleep problems, as well as the protective role of physical activity for both issues (Biddle et al., 2019), no study has investigated how these problems reciprocally interact from adolescence to early adulthood and whether physical activity plays a protective role in this interaction. Furthermore, there is a lack of understanding regarding potential gender differences in these developmental interactions. This study aims to address these gaps by examining the bidirectional associations among anxiety symptoms, sleep problems, and physical activity using a longitudinal community sample spanning from early adolescence to early adulthood, while also exploring gender differences in this development.

There is a vast literature that links poor sleep quality to mental health problems. Poor sleep quality, including short sleep duration, low efficiency, sleep fragmentation, and wake-after-sleep-onset, in adolescence has been associated with increased depressive symptoms, suicidal thoughts, self-harm behaviors, cognitive impairments (Gradisar et al., 2022; Kuula et al., 2015; Liu et al., 2017), and anxiety symptoms (Narmandakh et al., 2020), with the last being the focus of this study. As regards possible explanations for this link, the theory of the cognitive model of insomnia postulates that anxiety symptoms, including excessive worry and intrusive thoughts, maybe one of the factors provoking insomnia (Harvey, 2002). This theory is extended by hyperarousal model of insomnia that included also neurobiological factors, such as monoamides and cortisol (see Riemann et al., 2020 for a review). Some support has been found for these theories, especially in adults (Su et al., 2021). However, most of the studies conducted with adolescents suggest a bidirectional relation between sleep quality and anxiety over time (e.g., Alvaro et al., 2013; Geng et al., 2018; Shanahan et al., 2014; Tochigi et al., 2016), or a stronger influence of sleep quality on anxiety symptoms than the influence of anxiety on sleep (e.g., Kelly & El-Sheikh, 2014; Narmandakh et al., 2020; Roberts & Duong, 2017). Poor sleep during adolescence might reduce myelination of anterior white matter and connectivity in fronto-limbic circuits, areas that are involved in the evaluation and processing of negative emotions, making a person more likely to develop anxiety disorders (Jamieson et al., 2021). Therefore, regardless of if the association is bidirectional, sleep problems might precede the development of anxiety problems. Understanding whether one influences the other during specific time periods can be instrumental in developing effective programs to prevent anxiety and/or sleep problems. Moreover, adolescence seems to be an appropriate period to examine the bidirectional association between these two conditions, given that both problems develop and can be exacerbated during that time.

Physical activity has often been associated with both anxiety and sleep problems. First, there is consistent evidence that physical activity, assessed as frequency and/or duration of physical movement per week, including different forms, such as walking, doing team sport, swimming, has a positive effect on mental health (see Biddle et al., 2019; Spruit et al., 2016). However, while the positive effects on depressive symptoms are well-established (Biddle et al., 2019; Dale et al., 2019), studies that focus on anxiety are more contradictory and often of low quality (see Carter et al., 2021 for a review). There are some hypotheses, partly validated, regarding the mechanisms that lead from physical activity to anxiety. The most well-supported, thus far, are related to physiological and psychological mechanisms (see Anderson & Shivakumar, 2013 for a review; Kandola et al., 2018). Physiologically, physical activity is linked to lower hypothalamic-pituitary-adrenal axis reactivity (e.g., Rimmele et al., 2007), which plays an important role in adaptive responses to stressors and is related to both depression and anxiety (Landgraf et al., 1999). As regards psychological mechanisms, physical activity might reduce anxiety symptoms through reducing anxiety sensitivity (Broman-Fulks et al., 2004; Broman-Fulks & Storey, 2008), i.e., the tendency to misinterpret and exaggerate anxiety-related sensations. The physiological symptoms that are feared by a person with high anxiety levels are usually activated through physical activity and such exposure could help to increase tolerance of these symptoms and decrease anxiety levels when encountering stressful stimuli in an everyday context. Activation through exercise of the physiological reactions that are seen in both physical activity and anxiety, such as a rapid heart rate, would also help to increase self-efficacy related to mastery of such symptoms in everyday life as well (Petruzzello et al., 1991). To summarize, though the evidence is still scarce, there is good reason to connect physical activity to reduced anxiety problems.

Physical activity has also been found to have a positive effect on sleep. Recent reviews have found that adults who do more exercise have a better sleep quality (Huang et al., 2023; Kline et al., 2021). Similar effects have been found for adolescents, although evidence is scarce (Kline et al., 2021; Lang et al., 2013; Raudsepp, 2018) and not always confirmed (see Zhao et al. 2023 for a review). The mechanisms through which physical activity would improve sleep quality are unclear, but two main hypotheses are currently advocated. The first focuses on physical activity-promoted physiological changes that can improve sleep regulation. This hypothesis has found support in some studies, with higher levels of exercise increasing the effect on sleep (Dworak et al., 2008; Kalak et al., 2012). Another hypothesis focuses on psychological mechanisms, postulating that physical activity improves sleep quality through decreasing anxiety and depressive symptoms (Biddle & Mutrie, 2007). The latter hypothesis got some support from the aforementioned studies showing a link between physical activity and mental health (Biddle et al., 2019) and is also supported by the fact that mental health problems and sleep often act on similar physiological processes (Baglioni et al., 2016).

Despite the well-documented relations between physical activity, anxiety, and sleep quality, very few studies have considered all three aspects together. Among them is a study with college students, which found that high and moderate levels of physical activity and sleep quality were both negatively associated with anxiety (Ghrouz et al., 2019). Also, the interactive effects of sleep duration and physical activity on adolescents’ anxiety were examined by Ogawa et al. (2019), who found that either adequate sleep or adequate physical activity predicted lower anxiety symptoms. Lastly, Zhu et al. (2019) found that fulfilling three 24 h movement guidelines, including physical activity and sound sleep, was related to decreased risk of anxiety in children and adolescents. However, all these studies were cross-sectional and therefore could not investigate the directions of the relations. This is problematic, as there is evidence of longitudinal reciprocal relations over time between anxiety and sleep (e.g., Geng et al., 2018; Narmandakh et al., 2020; Kelly & El-Sheikh, 2014; Tochigi et al., 2016), and between physical activity and sleep (e.g., Master et al., 2019; Raudsepp, 2018). Nevertheless, to the best of our knowledge, no study has examined how these three factors interact over time in adolescence.

Another limitation of the aforementioned studies, with the exceptions of few studies (Tochigi et al., 2016; Narmandakh et al., 2020), is their inability to disentangle the roles of stable, between-person differences and within-person changes. This distinction is crucial for developing personalized interventions, as conclusions drawn at the between-person level may not necessarily apply to within-person dynamics. For example, many studies investigating reciprocal relations rely on Cross-Lagged Panel Models (CLPM). However, a limitation of these models is their assumption of no stable intra-individual differences in variables. This assumption is inaccurate for psychological constructs such as anxiety and sleep, which tend to exhibit trait-like characteristics and maintain some level of stability in between-person rank order. Therefore, to understand how changes in sleep at one assessment impact anxiety at the next assessment, and vice versa, it is essential to distinguish between between-person and within-person effects. Unfortunately, very few studies utilize statistical models that effectively separate within-person effects from between-subjects (as seen in Narmandakh et al., 2020), and none have adopted this approach to investigate the interaction among physical activity, sleep problems, and anxiety symptoms.

Additionally, the development of sleep problems, anxiety, and physical activity varies between girls and boys. For example, girls and boys exhibit differences in the development of sleep habits from childhood to adolescence (e.g., Laberge et al., 2001) and from adolescence to early adulthood (e.g., Saelee et al., 2023), possibly due to variations in puberty timing (e.g., Laberge et al., 2001). Furthermore, both anxiety symptoms and physical activity not only differ in rates between girls and boys but also show distinct developmental trajectories throughout adolescence. For instance, the general decline in physical activity is more pronounced for girls than boys in early adolescence, whereas the trend reverses in late adolescence (Dumith et al., 2011). Moreover, anxiety symptoms tend to be higher in girls than boys and exhibit different trajectories and correlates throughout adolescence (Letcher et al., 2012). Given these developmental differences, it is reasonable to assume that the interactions among anxiety symptoms, sleep problems, and physical activity might differ between girls and boys. Consistent with this notion, differences in the relationships between physical activity and anxiety symptoms between girls and boys have also been observed (Buchan et al., 2021). To the best of our knowledge, no study exists that investigates how sleep problems, anxiety symptoms, and physical activity interact over time differently in boys and girls.

## Current Study

There is a lack of knowledge regarding the reciprocal interaction between anxiety symptoms and sleep problems during adolescence. Furthermore, limited research has been conducted to explore whether physical activity could potentially influence this relationship. This study seeks to address this gap by investigating longitudinal bidirectional relations between physical activity, sleep problems, and anxiety symptoms from early adolescence to early adulthood, employing a statistical approach, i.e., RI-CLMP, that enables to differentiate between variations among individuals (between-person) and variations within individuals (within-person). Based on the aforementioned theoretical frameworks, a positive reciprocal relation between sleep problems and anxiety symptoms across adolescence is expected. Moreover, it is expected that physical activity would act as protective factor and decreases both anxiety symptoms and sleep problems. Differences between boys and girls in these relations across adolescence will be also tested.

## Method

### Participants and Procedure

The data came from the “SALVe cohort” project, which has investigated the psychological and psychosocial determinants in two groups, born in 1997 and 1999, and followed them from childhood to adulthood. All individuals born in 1997 and 1999 and living in the Swedish region of Västmanland in 2012 were contacted for the study. However, some youth (such as those who had lived in Sweden for less than five years and those suffering from mental illness or serious disease) were excluded due to inclusion criteria (see Vadlin et al., 2015 for more details). In the present study, we focused on the individuals born in 1999, as they had complete data covering the period from early adolescence to early adulthood (from 13 to 22 years old). The adolescents were contacted when they were 12–13 years (T1 early adolescence), 15–16 years (T2 middle adolescence), 18–19 years (T3 late adolescence), and 21–22 years (T4 early adulthood). At T1, they were contacted by post and invited to participate in the longitudinal study. They were informed that their participation was voluntary and that they could leave the study at any time. Those willing to participate returned a self-reported questionnaire by post at T1 and T2. At T3 and T4, the questionnaire was filled out online. At T1, both parents and adolescents gave written consent for participation, while at T2, T3, and T4 only the adolescents gave consent, in accordance with Swedish law.

**Fig. 1:**
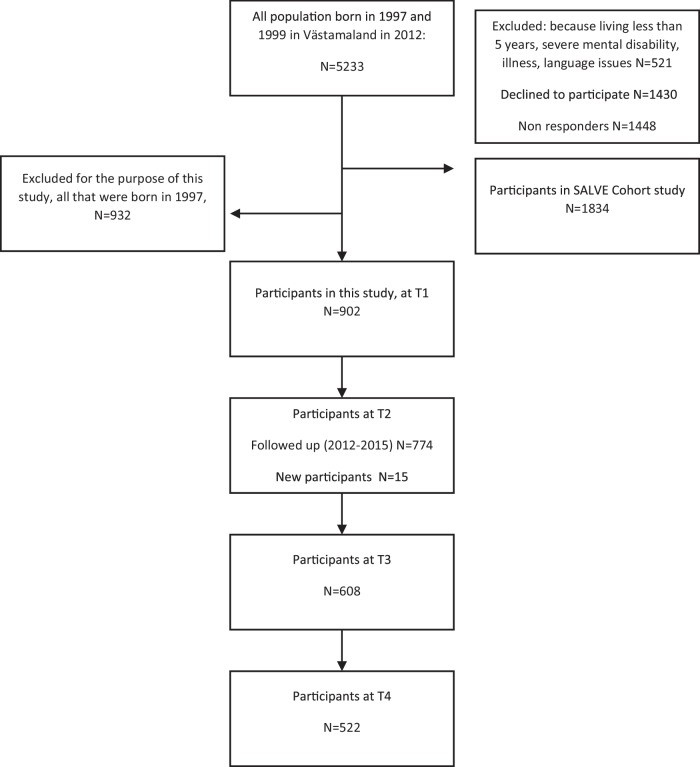
Flowchart of the study population

A questionnaire, exploring different psychological and social constructs, such as adolescents' internalizing and externalizing problems, gambling, gaming, social media use, relations with parents and peers, and school involvement was sent out to 2289 young people (born in 1999) in wave 1 (T1), of whom 935 (about 41%) responded (see Fig. 1). The final sample responding to at least one questionnaire consisted of 915 young people, of whom 57% (*N* = 475) were girls; 21% (*N* = 171) had non-Scandinavian born parents. The sample is representative of the broader Swedish adolescents population in terms of proportions of separated parents (30%), single-parent households (19%), and employed parents (92%) (see Olofsdotter et al., 2018).

### Measures

#### Frequency of vigorous physical activity

Physical activity was assessed with a measure of vigorous physical activity, as there is some evidence that high-intensity physical activity has greater effects in reducing sleep problems (Feng et al., 2014) and anxiety symptoms (Aylett et al., 2018; Azevedo Da Silva et al., 2012). Vigorous physical activity was measured with the item: “How often do you exercise for at least 30 min in your spare time so that you get tired/sweaty?”. This is a slightly modified item from the Health Behavior in School-aged Children (HBSC) study (see HBSC for more information); we added the indication of activity duration (i.e., “for at least 30 min”), to improve the reliability of the measure. The original item has been validated in international studies and showed good reliability and fair validity (Booth et al., 2001; Rangul et al., 2008). The response options ranged from “never” (0) to “every day” (6).

#### Anxiety symptoms

Anxiety symptoms were measured using the Generalised Anxiety Disorder subscale of the more comprehensive Spence Children’s Anxiety Scale (SCAS Spence, 1997) at T1 and T2, whereas a GAD subscale from the Adult Anxiety Scale (AAS-15; Hedqvist et al. 2023) was used at T3 and T4. The SCAS GAD subscale consists of the following items: (1) “I worry about things”; (2) “When I have a problem, I get a funny feeling in my stomach”; (3) “I feel afraid”; (4) “When I have a problem, my heart beats really fast”; (5) “I worry that something bad will happen to me”; (6) “When I have a problem, I feel shaky.” Response options range from 0 (never) to 3 (always). The AAS-15 GAD subscale, used at T3 and T4, consists of the following items: (1) “I worry about things a great deal”; (2) “I worry a lot more than most people”; (3) “I worry about all sorts of different things”; (4) “I find it difficult to stop worrying”; (5) “I feel tense or irritable.” Cronbach’s alphas were 0.77 at T1 (male = 0.73 and female = 0.77), 0.83 at T2 (male = 0.77 and female = 0.82), 0.92 at T3 (male = 0.89 and female = 0.91), and 0.92 at T4 (both male and female = 0.92).

#### Sleep problems

Sleep problems were assessed with the Karolinska Sleep Questionnaire (KSQ, Nordin et al., 2013), which includes three parts assessing dimensions of sleep quality (four items, e.g., difficulties falling asleep, repeated awakenings with difficulties falling asleep again, premature awakenings, disturbed/restless sleep), non-restorative sleep (five items, e.g., difficulties waking up, not well-rested on awakening, feelings of being exhausted at awakening, sleepiness at school or at work, sleepiness during leisure time), and sleep apnea (three items, heavy snoring, gasping for breath during sleep, cessation of breathing during sleep). Response options range from “never” (0) to “5 times or more per week” (5). Higher scores correspond to a higher number of problems. Cronbach’s alphas were 0.84 at T1 (male = 0.83 and female = 0.86), 0.88 at T2 (male = 0.85 and female = 0.88), 0.88 at T3 (male = 0.86 and female = 0.89), and 0.89 at T4 (male = 0.88 and female = 0.91), respectively.

#### Covariate and moderator

Sex (boy or girl) was retrieved from the participant’s social security number and used as a moderator. The birth country of the parents was assessed with two questions regarding where the mother and father, respectively, were born. Possible answers were: Sweden, Nordic country, another country in Europe, and outside Europe. These items were combined into one item, dichotomized as Scandinavian parents or at least one parent born outside Scandinavia, and used as a covariate in the analyses.

### Plan of Analyses

To achieve our aims, a random intercept cross-lagged panel model (RI-CLPM) was used. The RI-CPLM is an extension of the more common cross-lagged panel model (CLPM), which has recently been criticized for getting between-person and within-person effects mixed up (Hamaker et al., 2015), leading to biased conclusions. While RI-CLPM distinguishes between a between-person time-invariant variance and variance due to within-person fluctuations (e.g., whether a youth who reported higher than average sleep problems at T1 had more anxiety symptoms at T2 than at T1). This is particularly important when considering psychological factors that might have a trait-like component, like anxiety and sleep quality (e.g., Saviola et al., 2020). In order to use the model, first three latent factors, representing sleep quality, vigorous physical activity, and anxiety symptoms, respectively, were created. The observed factors of sleep quality, vigorous physical activity, and anxiety symptoms, were constrained to 1 in each respective latent factor. The residual variances of the observed variables were also constrained to 0, which enabled the latent factor structure to capture the within- and between-person variance. At the within-person level, autoregressive and cross-lagged paths were estimated among all twelve latent factors. A significant autoregressive path would indicate that an individual change higher or lower than expected for a certain factor at one timepoint would predict a greater increase or decrease than expected for the same factor at the following timepoint. A significant cross-lagged path between T1 and T2 would indicate that a mean higher than the expected individual mean at T1 would predict a greater increase at T2. Moreover, a significant cross-lagged path from T2 to T3 and from T3 to T4 would indicate that a smaller/larger individual change than expected in one factor at one timepoint would predict a larger/smaller change than expected in the other factor at the following timepoint. At the between-person level, correlations between the random intercepts indicate stable differences between youth (e.g., whether youth who sleep better than their peers also do more physical activity and show less anxiety symptoms). In the model, parents' country of birth was used as a proxy for immigration status as this is a relevant factor for mental health (see Close et al., 2016), and was treated as a time-invariant constant and regressed on all the observed variables (Hamaker, 2018).

In order to investigate whether the relations between sleep quality, vigorous physical activity, and anxiety symptoms were the same over time for girls and boys, a multigroup model was performed. First, cross-lagged paths were left free and then constrained to be identical for girls and boys. Then, the chi-squared values from the constrained model and the non-constrained models were compared. A non-significant difference in chi-squared values would mean that the two groups did not differ with regard to the relations examined.

In all models, missing data were imputed using the full information maximum likelihood (FIML) method in Mplus (Muthén & Muthén, 1998–2012). This procedure is recommended when data are missing at random or missing completely at random (Little & Rubin, 2019) and are longitudinal (Little, 2013), as it provides better estimates than pair- or list-wise deletion. With regard to indices of model fit, chi-squared values, the root mean square error of approximation (RMSEA) value, and the comparative fit index (CFI) are reported. The RMSEA value, which is a measure of approximate fit in the population, is good when <0.05, and acceptable at 0.05–0.10 (Hu & Bentler, 1998). Lastly, the CFI (Hu & Bentler, 1998) should be above 0.95.

## Results

### Descriptives

Correlations between all the variables and their means and standard deviations are presented in Table 1. All the correlations had the expected directions, with negative correlations between vigorous anxiety symptoms and anxiety symptoms and between vigorous physical activity and sleep problems, and positive correlations between anxiety symptoms and sleep problems at all timepoints.

**Table 1:** Correlations, means, and standard deviations (SDs) for sleep problems (SLE), generalized anxiety disorder (GAD), and vigorous physical activity (VPA) across all waves

Correlations	1.	2.	3.	4.	5.	6.	7.	8.	9.	10.	11.	12.
1. SLE T1												
2. GAD T1	0.45^**^											
3. VPA T1	−0.16^**^	−0.05										
4. SLE T2	0.41^**^	0.34^**^	−0.09^*^									
5. GAD T2	0.25^**^	0.46^**^	−0.05	0.55^**^								
6. VPA T2	−0.14^**^	−0.13^**^	0.43^**^	−0.11^**^	−0.14^**^							
7. SLE T3	0.35^**^	0.27^**^	−0.07	0.52^**^	0.37^**^	−0.09^*^						
8. GAD T3	0.22^**^	0.33^**^	−0.07	0.31^**^	0.47^**^	−0.19^**^	0.47^**^					
9. VPA T3	−0.11^**^	−0.05	0.27^**^	−0.14^**^	−0.12^**^	0.46^**^	−0.16^**^	−0.14^**^				
10. SLE T4	0.23^**^	0.18^**^	−0.06	0.42^**^	0.28^**^	−0.06	0.46^**^	0.22^**^	−0.13^**^			
11. GAD T4	0.17^**^	0.26^**^	−0.06	0.33^**^	0.44^**^	−0.14^**^	0.33^**^	0.59^**^	−0.12^**^	0.45^**^		
12. VPA T4	−0.11^**^	−0.11^*^	0.28^**^	−0.06	−0.16^**^	0.39^**^	−0.04	−0.16^**^	0.52^**^	−0.12^**^	−0.18^**^	
Descriptives												
Range	0–60	0–18	0–6	0–60	0–18	0–6	0–60	0–15	0–6	0–60	0–15	0–6
1. Means	10.04	3.31	3.85	15.95	4.96	3.89	18.84	5.33	3.01	19.61	6.40	3.05
2. SD	7.9	2.67	1.50	9.96	3.54	1.64	10.85	4.23	1.79	10.52	4.37	1.68

*SLE* sleep problems, *GAD* generalized anxiety disorder symptoms, *VPA* vigorous physical activity, *T1* data collected at time 1, early adolescence, *T2* data collected at time 2, middle adolescence, *T3* data collected at time 3, late adolescence, *T4* data collected at time 4, early adulthood

*p < 0.05 **p < 0.001

At T1, 6% (*N* = 51) of the adolescents were physically inactive, a figure that rose to 7% (*N* = 53) at T2 and to 11% (*N* = 69) at T3 and T4. Mean levels for anxiety symptoms rose from 3.31 (SD = 2.67) at T1 to 4.96 (SD = 3.54) at T2 and from 5.33 (SD = 4.23) at T3 to 6.40 (SD = 4.37) at T4. Finally, also mean levels of sleep problems increased from 10.09 (SD = 7.9) at T1 to 19.62 (SD = 10.61) at T4.

### Attrition Analyses

Attrition analyses were conducted to investigate whether adolescents with high anxiety symptoms, sleep problems, and low vigorous physical activity were more likely to drop out from the study after T1. The means at T1 of adolescents who dropped out at T2, T3, and T4 versus those that did not, were compared. No differences in anxiety symptoms (*M* = 3.30, SD = 2.70 vs *M* = 3.39, SD = 2.48, respectively, *F* (1, 895) = 0.14, n.s.), sleep problems (*M* = 9.83, SD = 7.42 vs *M* = 11.17, SD = 9.24, respectively, *F* (1, 897) = 3.15, n.s.), and vigorous physical activity (*M* = 3.11, SD = 1.46 vs *M* = 3.35, SD = 1.71, respectively, *F* (1, 893) = 2.81, n.s.), was found at T1 between those that dropped out at T2 and those that did not. Those that dropped out at T3 showed more sleep problems at T1 compared to those that did not drop out (*M* = 11.03, SD = 8.49 vs *M* = 9.51, SD = 7.50 respectively, *F* (1, 897) = 7.53, *p* < 0.05), while there were no differences for anxiety symptoms (*M* = 3.37, SD = 2.80 vs *M* = 3.28, SD = 2.60, respectively, *F* (1, 895) = 0.23, n.s.), and vigorous physical activity (*M* = 3.10, SD = 1.53 vs *M* = 3.17, SD = 1.49, respectively, *F* (1, 893) = 0.41, n.s.). Additionally, those that did not participate at T4 showed more sleep problems at T1 compared to those that did participate (*M* = 10.73, SD = 8.19 vs *M* = 9.48, SD = 7.60, respectively, *F* (1, 897) = 5.58, *p* < 0.05), while there were no differences for anxiety symptoms (*M* = 3.38, SD = 2.77 vs *M* = 3.26, SD = 2.59 respectively, *F* (1, 895) = 0.43, n.s.), and vigorous physical activity (*M* = 3.38, SD = 2.78 vs *M* = 3.26, SD = 2.59 respectively, *F* (1, 893) = 0.17, n.s.).

### Reciprocal within- and between-Person Associations between Vigorous Physical Activity, Sleep Problems, and Anxiety Symptoms

The standardized model coefficients of RI-CLPM are summarized in Table 2. The model had a good fit (*χ*^2^ = 44.11, df = 21, *p* < 0.05, CFI = 0.99, RMSEA = 0.035, C.I = 0.020–0.049).

**Table 2:** Parameter standardized estimates for RI-CLPM linking sleep problems (SLE), generalized anxiety disorder (GAD) symptoms, and vigorous physical activity (VPA)

Parameters	*B*	SE	*p*	*B*	SE	*p*	*B*	SE	*p*	*B*	SE	*p*
Cross-lagged path	T1→T2			T2→T3			T3→T4					
SLE- > VPA	−0.027	0.06	n.s.	−0.07	0.06	n.s.	0.13	0.05	0.04			
SLE- > GAD	0.011	0.06	n.s.	0.10	0.05	0.04	0.08	0.05	0.09			
GAD- > VPA	−0.041	0.07	n.s.	−0.03	0.06	n.s.	−0.14	0.05	0.01			
GAD- > SLE	0.134	0.07	0.04	0.12	0.06	0.02	−0.02	0.05	n.s.			
VPA- > SLE	−0.03	0.05	n.s.	0.01	0.05	n.s.	0.09	0.05	0.02			
VPA- > GAD	0.021	0.05	n.s.	−0.14	0.05	0.01	−0.08	0.05	0.09			
Stability paths												
SLE	0.116	0.07	0.09	0.34	0.05	0.01	0.35	0.05	0.01			
GAD	0.177	0.09	0.05	0.29	0.06	0.01	0.48	0.06	0.01			
VPA	0.175	0.06	0.01	0.29	0.05	0.01	0.38	0.05	0.01			
Correlation/ Correlated change		T1			T2			T3			T4	
SLE with GAD	0.26	0.08	0.01	0.48	0.04	0.01	0.35	0.08	0.01	0.39	0.04	0.01
SLE with VPA	−0.05	0.06	n.s.	−0.02	0.05	n.s.	−0.09	0.04	<0.05	−0.05	0.05	n.s.
GAD with VPA	0.09	0.08	n.s.	−0.09	0.05	0.09	−0.06	0.04	n.s.	−0.09	0.05	0.04
Between- person	CORRELATIONS	ACROSS	WAVES									
SLE with GAD	0.70	0.08	0.01									
SLE with VPA	−0.32	0.11	0.01									
VPA with GAD	−0.26	0.11	0.01									

*SLE* sleep problems, *GAD* generalized anxiety disorder symptoms, *VPA* vigorous physical activity, *T1* data collected at time 1, early adolescence, *T2* data collected at time 2, middle adolescence, *T3* data collected at time 3, late adolescence, *T4* data collected at time 4, early adulthood, *n.s.* non-significant

At the between-person level, sleep problems were positively associated with anxiety symptoms, suggesting that adolescents who had higher scores for sleep problems across all four waves also reported more anxiety symptoms (*r* = 0.70, SE = 0.08, *p* < 0.01). Conversely, vigorous physical activity was negatively associated with both sleep problems (*r* = −0.32, SE = 0.11, *p* < 0.05) and anxiety symptoms (*r* = −0.26, SE = 0.11, *p* < 0.05), indicating that youth with higher frequency of vigorous physical activity had fewer sleep problems and anxiety symptoms throughout adolescence.

At the within-person level, cross-sectional associations between vigorous physical activity and anxiety symptoms (*r* = 0.09, n.s.) and vigorous physical activity and sleep problems (*r* = −0.05, n.s.) were not significant at T1 (early adolescence), while anxiety symptoms and sleep problems correlated positively (*r* = 0.26, *p* < 0.01). Anxiety symptoms and sleep problems correlated positively at T2 (middle adolescence, *r* = 0.48, *p* < 0.01), T3 (late adolescence, *r* = 0.36, *p* < 0.01), and T4 (early adulthood, *r* = 0.39, *p* < 0.01), indicating that adolescents who reported more anxiety symptoms than their average at a specific timepoint also reported more sleep problems than their average at that timepoint. Vigorous physical activity at T3 (late adolescence) was negatively correlated with sleep problems at T3 (*r* = −0.09, *p* < 0.05), indicating that adolescents who reported more vigorous physical activity than their average at T3 reported fewer sleep problems than their average at that timepoint. Lastly, vigorous physical activity at T4 (early adulthood) was negatively correlated with anxiety symptoms at the same timepoint (*r* = −0.09, *p* < 0.05), suggesting that adolescents who reported more vigorous physical activity than usual at T4 also showed fewer anxiety symptoms than usual at the same time point. The other cross-sectional links were not significant.

**Fig. 2:**
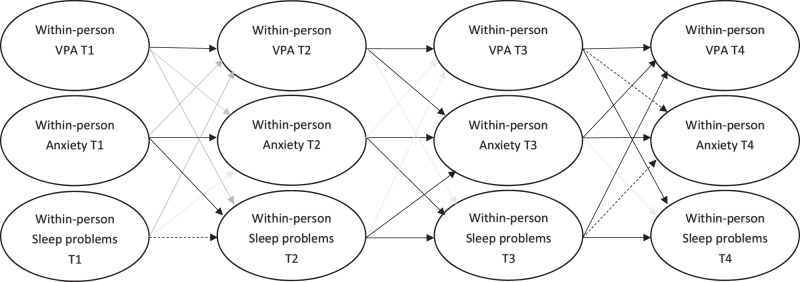
Depiction of cross-lagged paths of random intercept cross-lagged panel model indications of within-person effects. Solid, black lines indicate significant effects (*p* < 0.05). Solid, grey lines indicate non-significant effects. Dashed lines indicate marginally significant paths (*p* < 0.10). VPA vigorous physical activity, T1 data collected at time 1, early adolescence, T2 data collected at time 2, middle adolescence, T3 data collected at time 3, late adolescence, T4 data collected at time 4, early adulthood

All the autoregressive paths were significant, except for the autoregressive stability path for sleep from T1 to T2 that was only marginally significant (Beta = 0.12, SE = 0.07, *p* < 0.10) (see Table 2 and Fig. 2), indicating that deviations from the individual average score in a factor were always related to an unexpected change in the same factor at the following timepoint. As regards within-person cross-lagged paths, anxiety symptoms at T1 (early adolescence) predicted sleep problems at T2 (middle adolescence), Beta = 0.13, SE = 0.07, indicating that higher scores in anxiety than average (i.e., higher than individual mean levels) at T1 (early adolescence) predicted a greater than expected increase (i.e., higher than individual change mean levels) in sleep problems at T2 (middle adolescence). The other cross-lagged paths from T1 to T2 were not significant (see Fig. 2 and Table 2).

Vigorous physical activity (Beta = −0.14, SE = 0.05, *p* < 0.01) and sleep problems (Beta = 0.10, SE = 0.05, *p* < 0.05) at T2 predicted anxiety symptoms at T3. Specifically, a greater than expected increase in vigorous physical activity at T2 (middle adolescence) predicted a greater than expected decrease in anxiety symptoms at T3 (late adolescence). Conversely, a greater than expected increase in sleep problems at T2 (middle adolescence) predicted a greater than expected increase in anxiety symptoms at T3 (late adolescence). In addition, a greater than expected increase in anxiety symptoms at T2 (middle adolescence) predicted a greater than expected increase in sleep problems at T3 (middle adolescence, Beta = 0.13, SE = 0.05, *p* < 0.05). The other cross-lagged paths from T2 to T3 were not significant (see Fig. 2 and Table 2).

As regards relations between T3 (late adolescence) and T4 (early adulthood), a greater than expected increase of sleep problems at T3 predicted a greater than expected increase in vigorous physical activity (Beta = 0.12, SE = 0.05, *p* < 0.05), and in anxiety symptoms at T4, though this last effect was only marginally significant (Beta = 0.08, SE = 0.05, *p* < 0.10). Nevertheless, a greater than expected increase of vigorous physical activity at T3 predicted a greater than expected decrease in sleep problems at T4 (Beta = −0.10, SE = 0.05, *p* < 0.05), and a slightly significant, greater than expected decrease in anxiety symptoms at T4 (Beta = −0.08, SE = 0.05, *p* < 0.10).

### Moderating Effects of Sex

Multigroup analyses were performed to investigate whether the associations were the same for girls and boys. The constrained model, where all the cross-lagged paths were constrained to be equal for girls and boys (chi-squared = 114.635, df = 57, RMSEA = 0.047, CFI = 0.97), did not differ from the unconstrained model (chi-squared = 98.435, df = 45, RMSEA = 0.05, CFI = 0.97, chi-squared = 16.2, df = 12, n.s.), indicating that the associations between vigorous physical activity, anxiety symptoms, and sleep problems did not differ between girls and boys.

## Discussion

There is a lack of understanding regarding the bidirectional within-person associations between anxiety symptoms and sleep problems during their development, particularly in the adolescent years, as well as the role of physical activity in this process. To bridge this gap, the current study tracked a cohort of early adolescents into early adulthood and aimed to investigate the bidirectional relations between sleep problems, vigorous physical activity, and anxiety symptoms, distinguishing between within-person and between-person sources of associations. Moreover, it was also investigated whether the relations between the aforementioned factors were the same for girls and boys. At the between-person level, associations between sleep problems, physical activity, and anxiety symptoms were bidirectional, that is, adolescents with higher scores for sleep problems also showed higher scores for anxiety symptoms and lower scores for vigorous physical activity. However, when looking at within-person associations, the picture became more complex, and sleep problems, anxiety symptoms, and vigorous physical activity showed a complex interrelation throughout adolescence, with an intensification of reciprocal interactions between middle and late adolescence. Moreover, these interrelations did not differ for girls and boys.

The first important finding of this study was that sleep problems and anxiety symptoms had a fairly reciprocal association, especially in middle adolescence, where deviations from the expected mean in one factor are associated with deviations from the expected change in the other factor 3 years later. Bidirectionality between sleep problems and anxiety symptoms has been detected in other studies conducted at the between-person level (Geng et al., 2018; Kelly & El-Sheikh, 2014; Shanahan et al., 2014; Tochigi et al., 2016). At the within-person level, results from this study showed that bidirectionality was evident only from middle to late adolescence and that anxiety symptoms drove the relation at earlier ages. This is in contrast with most past studies, which have indicated that sleep problems precede an increase in anxiety symptoms (e.g., Kelly & El-Sheikh, 2014). However, studies that investigate the topic from a developmental perspective, by following adolescents from the same age, have highlighted that sleep problems precede anxiety symptoms during a period between ages from 10 to 13 years old, i.e., the very beginning of puberty (Kelly & El-Sheikh, 2014; Narmandakh et al., 2020). It has been hypothesized that the onset of puberty might be a sensitive period for the effects of sleep quality on emotional development (Kelly & El-Sheikh, 2014). However, in line with this study, this effect is not present from age 13 years to age 16 years (Narmandakh et al., 2020)—a developmental period during which an effect of anxiety on insomnia, not vice versa, has been discovered in another study (Johnson et al., 2006). This underscores the need to adopt a developmental perspective when investigating the reciprocal associations between sleep and anxiety problems, given that these aspects are not completely stable, but evolve and change during adolescence (Beesdo-Baum & Knappe, 2012).

Results from this study are slightly different from those of the only other study, to our knowledge, that examines within-person associations. While the effects of sleep problems were quite consistent between the studies, Narmandakh et al. (2020) did not find any effect of anxiety symptoms on sleep problems. This difference might be due to the different measures used to assess anxiety symptoms and the limited reliability of the measures used in the study by Narmandakh et al. Regardless, more studies are needed before definitive conclusions are drawn.

Another important result is related to the role of vigorous physical activity in preventing anxiety symptoms. Physical activity interventions have been related to improvement in mental health, specifically depression (Biddle et al., 2019), while evidence regarding anxiety is scarce (Carter et al., 2021). Moreover, reciprocal associations between physical activity and anxiety have been found over time in studies with adults (Azevedo Da Silva et al., 2012; Hiles et al., 2017), though studies in adolescents have not confirmed this bidirectionality (Buchan et al., 2021; Gunnell et al., 2016). Again, adding a developmental perspective, our study extends this knowledge by adding an important point, which is that the effect of vigorous physical activity might precede the effect of anxiety symptoms. We found that a greater than expected increase of vigorous physical activity in middle adolescence might predict a greater than expected decrease of anxiety symptoms in late adolescence when anxiety symptoms become more important in driving the relation. The importance of middle adolescence for the protective effect on vigorous physical activity on mental health has been confirmed in a recent study from our group dealing with depressive symptoms (Giannotta et al., 2023). Adolescence is a period characterized by a decline in physical activity (Dumith et al., 2011) but our study suggests that physical activity-based interventions might succeed in decreasing anxiety symptoms.

One unexpected result was the relation between sleep problems and vigorous physical activity. First, we did not find any associations from early to late adolescence between sleep and vigorous physical activity. Evidence for longitudinal associations between sleep and physical activity in adolescence has been reported in several studies. For instance, a reciprocal association over one year between sleep disturbances and physical activity was reported in a study (Raudsepp, 2018), whereas another study found a bidirectional association between sleep offset and duration and moderate-to-vigorous physical activity in middle adolescence (Master et al., 2019). The results of these two studies are difficult to compare with those of this study, given the short time periods used: 1-year associations (Raudsepp, 2018) and day-to-day associations (Master et al., 2019). In support of this, another study (Antczak et al., 2020) revealed that while day-to-day associations between daytime physical activity and night-time sleep were significant, the effect almost disappeared in 2-year longitudinal associations. This calls for further studies taking account of all the different aspects of physical activity and sleep quality.

Second, while vigorous physical activity was negatively associated with sleep problems at the between-person level across all periods of investigation, a greater than expected increase in sleep problems in late adolescence predicted a greater than expected increase in vigorous physical activity in early adulthood at the within-person level. The effect was small and this result is difficult to interpret, but it is not the only controversial result found when looking at sleep and physical activity at the within-person level. For instance, two other studies (Krietsch et al., 2016; Master et al., 2019) found that an increase in night-time sleep duration predicted less moderate to vigorous physical activity the day after, while this association was reversed at the between-person level. To our knowledge, no study has investigated the reciprocal effects of vigorous physical activity and sleep disturbances in adolescence with a day-to-day design or with a within-person approach, which makes it difficult to speculate about the reasons for our unexpected result. Nevertheless, a study conducted on children using objective measures for both physical activity and sleep highlighted a positive bidirectional association between physical activity and poor sleep (Pesonen et al., 2011). Lastly, another study (Liao et al., 2020) found that having less sleep over two nights than one’s usual level predicted an increase in physical activity on the following day among a sample of overweight adults. This is very consistent with our results, given that sleep duration and sleep problems are associated (e.g., Chen et al., 2022). To summarize, the relation over time between physical activity and sleep appears to be more complex than expected and the investigation at the within-person level seems to suggest a J relation, implying that there is an optimal level of physical activity for improving sleep, something that has already been confirmed in adults. It is worth noting that a greater than expected increase of vigorous physical activity in late adolescence predicted a greater than expected decrease in sleep problems, despite this. Our study confirmed the protective role of physical activity in preventing sleep problems in adolescence, particularly late adolescence.

From a developmental perspective, this study underscores the significance of middle adolescence as a sensitive period for the emergence of a negative cycle between anxiety and sleep problems. The reasons for this association can only be hypothesized at this time. Middle adolescence, typically occurring between the ages of 14–15, is a period where changing socio-cultural factors may significantly influence adolescents’ lives. Regarding sleep, the transition to high school around age 14 poses a risk for increased sleep problems due to school-related stressors (Brodar et al., 2020). Additionally, adolescents, particularly in Sweden, have been observed to experience increased internalizing problems during this period, partly due to increased school stress and demands (Högberg et al., 2020). It could be hypothesized that the simultaneous increase in both anxiety and sleep problems during this time might intensify reciprocal associations, which may persist and escalate into late adolescence. Furthermore, environmental factors, such as those related to the school environment, may play a role in explaining this association. However, further research is needed to investigate whether these mechanisms contribute to the increase in reciprocal associations during middle adolescence.

No difference in the model between girls and boys were found. When it comes to vigorous physical activity and mental health, this result is consistent with our previous study focusing on depression and vigorous physical activity (Giannotta et al., 2023) but investigating the between levels effects. Conversely, another study found that moderate to vigorous physical activity predicted more anxiety symptoms one year later in girls, but not in boys (Buchan et al., 2021). To our knowledge, none of the studies that have investigated bidirectional associations between sleep, vigorous physical activity, and anxiety, distinguishing between- and within-person associations, has reported or investigated the moderating effect of sex, which makes us cautious in drawing conclusions about this. Nevertheless, the results of our study suggest that altering the average levels of anxiety symptoms, sleep problems, and physical activity, regardless of their initial levels, has similar effects in boys and girls. While evidence has suggested different developmental trajectories for boys and girls concerning sleep and anxiety levels (e.g., Saeele et al., 2023), as well as varying declines in physical activity throughout adolescence, our study implies that despite potential differences in the development of these problems, often influenced by biological and sociocultural factors (Saeele et al., 2023), the interrelations among them remain consistent. Deviations from the average levels of one construct appear to impact the others similarly. If confirmed by further studies, these results may suggest a similar approach for addressing anxiety and sleep problems in both girls and boys.

This study has some limitations. One of them is using only a self-assessment of vigorous physical activity. However, the measure has shown validity compared with objective measures (Booth et al., 2001) and in a recent study could be distinguished from moderate to vigorous physical activity (Tanaka et al., 2017). Moreover, moderate to vigorous physical activity has been positively associated with adolescents’ mental health and is recommended by the WHO. Future studies should explore reciprocal effects of moderate to vigorous physical activity and anxiety in adolescence. Another limitation is using slightly different measures to assess anxiety symptoms at the different timepoints. Although this has the strength of assessing symptoms using measures adapted to participants’ developmental stages and making all questions relevant and appropriate for age, the adaptations might lead to some continuity problems and affect our results. Moreover, in this study, only one dimension of anxiety—namely generalized anxiety disorder symptoms—was considered. This choice was made to make anxiety symptoms at different ages more comparable. Nevertheless, anxiety disorders are complex and can involve different dimensions that can be more predominant in one period of life than another (e.g., separation anxiety), and that can be influenced in different ways by sleep problems and vigorous physical activity. Also, even whether the self-reported measure used to assess sleep problems in this study is well known and validated, a more objective measure would have strengthened the results. Another limitation is that adolescents with sleep problems at T1 were more likely to drop out from the study. Even whether our procedure to control for missing data has valid support for handling with these cases (Little & Rubin, 2019), it cannot be completely ruled out that this attrition did influence our results. Moreover, the study did not include some important time-varying covariates that might have affected the results, i.e., negative life events, chronotype, sleep duration, sleep timing, use of sleeping medication, family income. As RI-CPLM can account properly time-invariant but not time-varying confounders if not included in the model (Usami et al., 2019), further studies should consider adding more time varying confounders in the model. Moreover, the sample is representative of some regions in Sweden, and the generalizability of the results to other populations is uncertain. Lastly, the correlation nature of the research design prevents us for claiming causality. Further research should take all the aforementioned aspects into account.

This study also has some strengths. To our knowledge, it is the first study to longitudinally test interrelations between anxiety symptoms, vigorous sleep problems, and sleep problems, for over 12 years, finding clear associations between all of them. Moreover, it is also the first study to investigate gender differences, finding that the relations between these factors were the same for girls and boys. In addition, to our knowledge, it is one of the first studies able to disentangle within-person and between-person changes, thanks to the RI-CLP model. This is particularly important when examining relatively stable factors which might have a genetic component, such as sleep and anxiety. The RI-CLP model is also recommended over the classic CLP model when more than three data waves are used (see Orth et al., 2021).

This study holds several practical implications. Primarily, it is crucial to consider the practical implications in terms of prevention that may stem from results obtained using RI-CPLM over CPLM. CPLM provides insights into how adolescents with high levels of sleep problems, for example, maybe more prone to experiencing elevated levels of anxiety compared to their peers. Such findings could offer guidance for targeting high-risk youth with sleep or anxiety issues to mitigate risks during specific developmental stages. Consequently, conclusions drawn from CPL models could aid in designing interventions for adolescents at greater risk of adverse developmental outcomes relative to their peers. However, a portion of this risk level may be attributed to traits, particularly concerning psychosocial constructs like anxiety and sleep. Hence, for effective prevention program planning, understanding whether subjective changes in these traits correlate with higher or lower risks of negative outcomes is crucial. This is precisely where RI-CPL models contribute to existing knowledge. The study’s conclusions can shed light on the effectiveness of behavior modifications during specific developmental periods and guide preventive efforts accordingly. Regarding the study’s findings, they indicate that the period from middle to late adolescence is pivotal for implementing interventions to enhance adolescent mental health. Firstly, changes during this period in sleep problems and anxiety symptoms, regardless of their initial levels, are interconnected, suggesting that interventions targeting one could also impact the other. Secondly, and more significantly from a public health standpoint, interventions aimed at modifying levels of vigorous physical activity during middle to late adolescence, a period when physical activity tends to decline, could potentially improve mental well-being. Such interventions could reduce anxiety symptoms and slightly alleviate sleep problems among all adolescents, regardless of their initial levels of issues.

## Conclusion

Despite numerous studies demonstrating bidirectional associations between sleep problems, anxiety symptoms, and physical activity, none have comprehensively investigated their reciprocal relationships throughout the entire adolescent period. Understanding the dynamics of these associations, particularly whether physical activity acts as a protective factor against the development of anxiety and sleep problems in both girls and boys, remains a crucial gap in the literature. This study aimed to address this gap by focusing on within-person associations, enabling an exploration of the developmental periods during which changes in sleep and anxiety symptoms might be linked to changes in physical activity, regardless of the initial level of problems. The findings suggest that deteriorations in anxiety symptoms during early adolescence may trigger cascading effects on other symptoms, such as sleep problems, until middle adolescence. During this period, both anxiety symptoms and sleep problems may mutually influence each other, making it challenging to discern the primary driving force. Furthermore, an increase in physical activity appears to offer greater protective effects during middle adolescence, coinciding with a decline in physical activity levels. These findings, applicable to both boys and girls, underscore the importance of identifying the appropriate developmental period for interventions aimed at improving adolescents’ mental health.
